# Assessing EAU criteria for high‐risk upper tract urothelial carcinoma

**DOI:** 10.1002/bco2.70044

**Published:** 2025-11-11

**Authors:** Mahmoud Farzat, Sami‐Ramzi Leyh‐Bannurah, Mykyta Kachanov, Florian M. Wagenlehner

**Affiliations:** ^1^ Department of Urology, Pediatric Urology, and Andrology Justus‐Liebig University of Giessen Giessen Germany; ^2^ Department of Urology and Robotic Urology Diakonie Klinikum Siegen Siegen Germany; ^3^ Department of University Medical Center Hamburg ‐ Eppendorf Department of Urology Hamburg Germany; ^4^ Institute of Human Genetics University Medical Center Hamburg‐Eppendorf Germany

**Keywords:** LAD, RANU, upper urinary tract carcinoma (UTUC)

## Abstract

**Background and Objective:**

Preoperative EAU guideline criteria stratify Upper tract urinary carcinomas (UTUC) into low‐ and high‐risk groups. Depending on this classification, high‐risk patients may receive simultaneous lymphadenectomy (LAD) during robot‐assisted radical nephroureterectomy (RANU). This study examines the reliability of such stratification, focusing on oncological outcomes.

**Methods:**

In 60 patients with UTUC who underwent RANU, 38 were stratified as low‐risk (63%) and 22 as high‐risk (37%) based on preoperative EAU guideline criteria. LAD was performed solely in the high‐risk group.

**Results:**

In the low‐risk group, 19 patients (50%) had non‐muscle‐invasive (Ta, Tis‐T1) disease, 7 (18%) had muscle‐invasive, locally confined (T2) disease and 12 (32%) had locally advanced (T3/4) disease. In the high‐risk group, results were 11 (50%), 3 (14%) and 8 (36%), with no statistical differences observed. Follow‐up (6 to 60 months) showed no significant differences in cancer‐specific (p = 0.3) and overall survival (p = 0.8) between groups. Limitations: the small sample size and the retrospective nature. Conclusions and Clinical Implications: The current EAU guideline‐based preoperative criteria for UTUC risk stratification demonstrated limited accuracy in identifying high‐risk patients undergoing RANU, potentially excluding some patients from receiving LAD.

**Patient Summary:**

We think that improved preoperative staging and risk stratification tools are needed to better manage patients with upper urinary tract urothelial carcinoma undergoing surgical treatment.

## 1 INTRODUCTION

Upper tract urinary cancers (UTUCs) represent a diagnostic challenge, as approximately 60% exhibit invasive behaviour at diagnosis.^1^ Accurate and standardized preoperative staging and risk stratification are essential before radical nephroureterectomy (RNU). Current staging relies on retrograde ureteropyelography, urine cytology, CT imaging and facultative ureteroscopy (URS) if imaging and cytology are insufficient for diagnosis.^1^ However, despite recent reports of high diagnostic sensitivity and specificity of these methods in UTUC detection,^2^ assessment of local lymph node (LN) status remains limited.^3^ Consequently, lymphadenectomy (LAD) during RNU may improve pathological staging accuracy^4^ and influence oncological outcomes.^5^, ^6^ Therefore, standardization of LAD templates has been proposed,^7^, ^8^ although the optimal extent and LN yield remain debated. However, given the decreasing risk of LN involvement with lower tumour stages,^4^ current European Association of Urology (EAU) guidelines mandate LAD exclusively in high‐risk UTUC cases.^1^ However, our study highlights the limited concordance between preoperative EAU guideline‐based risk stratification and final pathological results, suggesting possible misclassification of patients who might benefit from more extensive surgical approaches, including LAD.

## 2 METHODS

Between July 2019 and July 2024, 60 consecutive patients with UTUC underwent robotic‐assisted nephroureterectomy (RANU). All procedures were performed by a single surgeon using the Da Vinci X® surgical system (Intuitive Surgical, Sunnyvale, CA, USA).

All patients were preoperatively stratified according to EAU guidelines criteria.^1^ Those with unifocal disease, tumour size <2 cm, absence of high‐grade cytology, low‐grade biopsy on URS and no signs of invasion on CT imaging were categorized as low‐risk UTUC. Conversely, patients with high‐grade cytology or biopsy, evidence of local invasion on CT, variant histology, multifocal disease, tumour size ≥2 cm, or hydronephrosis were classified as high‐risk UTUC. LAD, performed exclusively in high‐risk patients during RANU, followed a template‐based LAD approach: ipsilateral iliac LNs for mid or distal ureter tumours and hilar, para‐aortic and interaortocaval LNs for renal pelvis or proximal ureter tumours.

Postoperative 90‐day complications were assessed using the Clavien‐Dindo classification (CDC). Follow‐up was conducted according to EAU guidelines.^1^

Patient data were collected in an institutional database after informed consent. The study was approved by the ethics committees of the Westfalen‐Lippe Medical Association and the University of Münster (2023–500‐f‐S). Statistical analyses were performed using SPSS® v27. Categorical variables are reported as frequencies, and continuous variables as means. For matched‐pair analysis, parametric and non‐parametric variables are compared using the independent T‐test and Mann–Whitney U tests. A one‐way ANOVA was used for parametric numeric variables, and the Kruskal‐Wallis test was applied to nonparametric variables.

## 3 RESULTS

### 3.1 Preoperative characteristics

Of the 60 patients, 38 (63%) were classified as low‐risk and 22 (37%) as high‐risk UTUC (Table 1). Positive cytology was observed in 12 patients (20%), with high‐grade cytology indicative of high‐risk UTUC in only 4 cases. Biopsy was positive in 17 patients (28%), with no significant difference in the clinical stage between groups (p = 0.3). CT staging revealed signs of invasion in 12 patients (20%). Tumour diameter exceeded 2 cm on CT in 15 patients (25%), in 7 of these patients, the findings were confirmed by retrograde ureteropyelography. Tumours were located in the renal pelvis in 40 patients (67%) and in the ureter in 20 patients (33%), with no significant difference between groups (p = 0.5).

**TABLE 1:** Baseline characteristics and preoperative clinical and oncological parameters.

	Total (N = 60)	Low‐risk UTUC	High‐risk UTUC	p‐value
		N = 38 (63%)	N = 22 (37%)	
Age (years), mean	70	72	66	0.9
BMI (kg/m^2^), mean	31	30	33	0.8
ASA‐score				
1	16 (26,7)	9 (24)	7 (32)	
2	15 (25)	8 (21)	7 (32)	0.3
3	29 (28)	21 (55)	8 (36)	
Neoadjuvant Chemotherapy cisplatin‐Gemcitabine	2 (3)	2 (5)	0	
Anti‐coagulation				*0.3*
ASA	18	13 (34)	5 (23)	
NOAC	6	4 (10)	2 (9)	
**EAU guidelines Criteria**				
Positive Cytology	12 (20)	8 (21)	4 (18)	*1*
High grade	4 (33)	0	4 (100)	
Tumour location				0.5
Ureter	20 (33)	12 (32)	8 (36)	
Kidney pelvis	40 (67)	26 (68)	14 (64)	
Preoperative Histology				*0.3*
No Histology	43 (72)	29 (77)	14 (64)	
Tis	1 (1,6)	0	1 (5)	
Ta	12 (20)	8 (21)	4 (18)	
T1	3 (5)	1 (2)	2 (9)	
T2	0	0	0	
T3	1 (1,6)	0	1 (5)	
Invasion on CT‐Scan	12 (20)	0	12 (55)	
Tumour‐Size (over 2 cm)	15 (25)	0	15 (69)	

Categorical data are presented as numbers %, UTUC: Upper Urinary Tract Urothelial Cell Carcinoma, BMI: body mass index, ASA: American Association of Anesthesiology Morbidity Score, ASA: Acetylsalicylic Acid, NOAC: new oral anticoagulants.

### 3.2 Postoperative characteristics and oncological outcomes

No significant differences were observed between preoperatively classified low‐ and high‐risk groups regarding pathological tumour stage (p = 0.9; Table 2). Twelve initially low‐risk patients (32%) exhibited pT3–4 stage. Similarly, no significant differences in tumour grade were found between groups. Within the low‐risk UTUC group, 3 patients (8%) had no tumour present at final pathology, while 25 patients (66%) were classified as grade 3. No positive surgical margins were observed. In the high‐risk UTUC group, an average of 10 LNs per patient were removed, with LN‐metastasis found in 3 patients (14%). Postoperatively, 14 patients (23%) received chemotherapy and 2 patients (3%) underwent adjuvant check‐point inhibitor therapy, with no difference between the groups (p = 0.3).

**TABLE 2:** Intra‐ and postoperative results, oncological outcomes a complications.

	Total N = 60	Low‐risk	High‐risk	p‐value
		UTUC	UTUC	
		N = 38 (63%)	N = 22 (37%)	
Pathological tumour stage, n (%)*				
Non muscle invasive (Tis‐T1)	30 (50)	19 (50)	11 (50)	0.9
Muscle invasive locally confined (T2)	10 (16)	7 (18)	3 (14)	
Locally advanced (T3–4)	20 (33)	12 (32)	8 (36)	
Urothelial carcinoma grade*, n (%)				
0	3 (5)	3 (8)	0	
1	13 (22)	7 (18)	6 (27)	0.9
2	5 (8)	3 (8)	2 (9)	
3	39 (65)	25 (66)	14 (64)	
Postoperative Chemotherapy (yes. vs. none), n (%)				
Cisplatin‐Gemcitabine, n (%)	14 (23)	8 (21)	6 (27)	0.3
Initiation of CI therapy, n (%)	2 (3)	1 (2,6)	2 (9)	
Positive surgical margins (total), (%)	0	0	0	
Number of lymph nodes removed in patients, who received a lymphadenectomy, mean (SD)	10	0	10 (1–31)	
Number of patients, who had a lymphadenectomy and had metastases among the total number of surgically treated patients	3 (5)	0	3 (14)	
Length of hospitalization (days), mean	7.7	8.2	5.7	0.04
Transfusion rate, n (%)	5 (8)	5 (13%)	0	0.8
Total Complications, n (%)	16 (26)	10 (26)	6 (27)	0.8
**CDC I**	4 (6,6)	3	1	
**CDC II**	1 (1,6)	0	1	
**CDC IIIa**	1 (1,6)	1	0	
**CDC III b**	7 (12)	3	4	
**CDC VI**	1 (1,6)	1	0	
**CDC V**	2 (3,2)	2	0	
Readmissions, n (%)	6 (10)	2 (5)	4 (18)	0.1
Bladder Recurrence, n (%)	6 (10)	4 (10)	2 (9)	0.2
Distant metastasis, n (%)	7 (12)	3 (8)	4 (18)	0.2
Cancer‐specific survival, n (%)	55 (92)	36 (95)	19 (86)	0.3
Overall Survival, n (%)	51 (85)	32 (84)	19 (86)	0.8

Categorical data are presented as numbers (%), UTUC: Upper Urinary Tract Urothelial Cell Carcinoma. SD: standard deviation, CI: Check‐point inhibitor,

* according to WHO classification 1999. CDC: Clavien‐Dindo Classification.

Over a follow‐up period of 6 to 60 months, urothelial bladder cancer recurrence occurred in six patients (10%) and distant metastasis developed in seven patients (12%) with no statistically significant difference between groups (both p = 0.2). Cancer‐specific and overall survival did not differ significantly between groups (p = 0.3 and p = 0.8, respectively; Table 2).

Regarding patient safety, there were no significant differences in transfusion rates or complication rates according to CDC (both p = 0.8) (Table 2).

## 4 DISCUSSION

Our findings suggest that EAU guideline‐based risk stratification—relying on conventional staging methods such as cytology, retrograde ureteropyelography, CT imaging and in selected cases, URS with histological sampling—does not reliably predict the necessity of simultaneous LAD during RNU. In our cohort, 38 patients initially classified as low‐risk UTUC did not undergo LAD. Postoperatively, however, a considerable proportion experienced significant tumour upgrading and upstaging, with seven patients (18%) showing locally confined‐ pT2) and 12 patients (32%) exhibiting locally advanced disease (pT3–T4). Additionally, 25 patients (66%) within the low‐risk group demonstrated high‐grade carcinoma on final pathology, underscoring substantial tumour upgrading. These findings suggest that such patients might have benefited from LAD during RANU. Conversely, 11 patients (50%) initially categorized as high‐risk exhibited non‐muscle‐invasive carcinoma (Tis–T1) postoperatively, indicating that LAD might have been unnecessary in these cases. The absence of differences in cancer‐specific and overall survival between the groups further reflects this significant discordance between preoperative risk stratification and final pathology findings. Additionally, given that rates of blood transfusion and CDC‐classified complications were comparable between the groups, we hypothesize that LAD may safely be offered selectively to patients initially classified as low‐risk until more accurate staging modalities or improved risk‐stratification tools become available.

Several limitations should be acknowledged, including the study's retrospective nature and small sample size. Furthermore, the EAU high‐risk criteria include imaging findings of renal parenchymal invasion. Despite this, approximately 30% of clinically low‐risk patients in our series had pT3/4 disease. This indicates a problem with imaging that fails to identify renal parenchymal invasion accurately. A larger study involving other centres would be beneficial to confirm that the issue is not centre‐specific.

During the preparation of this work, the authors used Grammarly to improve the readability and language of the manuscript. After using this tool, the authors reviewed and edited the content as needed and took full responsibility for the content of the publication.

## AUTHOR CONTRIBUTIONS

**Mahmoud Farzat:** Conceptualization; methodology; software; validation; formal analysis; investigation; data curation; writing—original draft; writing—review and editing; visualization; supervision; project administration. **Sami‐Ramzi Leyh‐Bannurah:** Writing—review and editing. **Mykyta Kachanov:** Writing—review and editing. **Florian M. Wagenlehner:** Writing—original draft; writing—review and editing; supervision; project administration.

## CONFLICT OF INTEREST STATEMENT

The authors declare no conflicts of interests.

## Supporting information


**Table S1:** complications, readmissions and oncological long‐term outcomes.

## References

[1] Roupret M , Seisen T , Birtle AJ , Capoun O , Comperat EM , Dominguez‐Escrig JL , et al. European Association of Urology guidelines on upper urinary tract urothelial carcinoma: 2023 update. Eur Urol. 2023;84(1):49–64. 10.1016/j.eururo.2023.03.013 36967359

[2] Janisch F , Shariat SF , Baltzer P , Fajkovic H , Kimura S , Iwata T , et al. Diagnostic performance of multidetector computed tomographic (MDCTU) in upper tract urothelial carcinoma (UTUC): a systematic review and meta‐analysis. World J Urol. 2020;38(5):1165–1175. 10.1007/s00345-019-02875-8 31321509

[3] Pallauf M , D'Andrea D , König F , Laukhtina E , Yanagisawa T , Rouprêt M , et al. Diagnostic accuracy of clinical lymph node staging for upper tract urothelial cancer patients: a multicenter, retrospective, observational study. J Urol. 2023;209(3):515–524. 36475808 10.1097/JU.0000000000003085

[4] Xylinas E , Kluth L , Rieken M , Roupret M , Al Hussein Al Awamlh B , Clozel T , et al. External validation of the pathological nodal staging score in upper tract urothelial carcinoma: a population‐based study. Urol Oncol. 2017;35(1):33.e21–33.e26. 10.1016/j.urolonc.2016.07.022 PMC5576549 27816402

[5] Hakimi K , Carbonara U , Djaladat H , Mehrazin R , Eun D , Reese A , et al. Outcomes of lymph node dissection in Nephroureterectomy in the treatment of upper tract urothelial carcinoma: analysis of the ROBUUST registry. J Urol. 2022;208(2):268–276. 10.1097/JU.0000000000002690 35377778

[6] Inokuchi J , Kuroiwa K , Kakehi Y , Sugimoto M , Tanigawa T , Fujimoto H , et al. Role of lymph node dissection during radical nephroureterectomy for upper urinary tract urothelial cancer: multi‐institutional large retrospective study JCOG1110A. World J Urol. 2017;35(11):1737–1744. 10.1007/s00345-017-2049-x 28508102

[7] Dominguez‐Escrig JL , Peyronnet B , Seisen T , Bruins HM , Yuan CY , Babjuk M , et al. Potential benefit of lymph node dissection during radical Nephroureterectomy for upper tract urothelial carcinoma: a systematic review by the European Association of Urology guidelines panel on non‐muscle‐invasive bladder cancer. Eur Urol Focus. 2019;5(2):224–241. 10.1016/j.euf.2017.09.015 29158169

[8] Bobjer J , Gerdtsson A , Abrahamsson J , Baseckas G , Bergkvist M , Bläckberg M , et al. Location of retroperitoneal lymph node metastases in upper tract urothelial carcinoma: results from a prospective lymph node mapping study. Eur Urol Open Sci. 2023;57:37–44. 10.1016/j.euros.2023.09.010 38020529 PMC10658412

