# Supplementary material for: Assessing EAU criteria for high‐risk upper tract urothelial carcinoma
Source: BJUI Compass. 2025 Nov 11;6(11):e70044. doi: 10.1002/bco2.70044 (PMC12605949; doi:10.1002/bco2.70044)
Supplement: Supplementary file 1 — Table S1: complications, readmissions and oncological long‐term outcomes. [file BCO2-6-e70044-s001.docx]

**Supplementary Table 1: complications, readmissions and oncological long-term outcomes.**

|  | | Total  (n=60) | Low-Risk  UTUC  N=38 (63%) | High-Risk  UTUC  N=22 (37%) | p-Value |
| --- | --- | --- | --- | --- | --- |
| Total Complications, n (%) | | 16 (26) | 10 (26) | 6 (27) | 0.8 |
| Minor | **CDC I** | 4 (6,6) | 3 | 1 |  |
|  | **CDC II** | 1 (1,6) | 0 | 1 |  |
| Major | **CDC IIIa** | 1 (1,6) | 1 | 0 |  |
|  | **CDC III b** | 7 (12) | 3 | 4 |  |
|  | **CDC VI** | 1 (1,6) | 1 | 0 |  |
|  | **CDC V** | 2 (3,2) | 2 | 0 |  |
| Readmissions, n (%) | | 6 10) | 2 (5) | 4 (18) | 0.1 |
| Bladder Recurrence, n (%) | | 6 (10) | 4 (10) | 2 (9) | 0.2 |
| Distant metastasis, n (%) | | 7 (12) | 3 (8) | 4 (18) | 0.2 |
| Cancer specific survival, n (%) | | 55 (92) | 36 (95) | 19 (86) | 0.3 |
| Overall Survival, n (%) | | 51 (85) | 32 (84) | 19 (86) | 0.8 |

Categorical data are presented as numbers (%), UTUC: Upper Urinary Tract Urothelial Cell Carcinoma, CDC: Clavien-Dindo Classicication(9).
